# Left Bundle Branch Area Pacing Prevents New-Onset Atrial Fibrillation and Improves Echocardiographic Parameters Compared with Right Ventricular Pacing in Patients with Bradyarrhythmias

**DOI:** 10.3390/biomedicines13061374

**Published:** 2025-06-04

**Authors:** Adrian-Ionuț Ungureanu, Georgică Târtea, Eugen Țieranu, Cristina Elena Negroiu, Gianina Cristiana Moise, Radu Mitruț, Victor Raicea, Radu-Gabriel Vătășescu, Paul Mitruț

**Affiliations:** 1 Doctoral School, University of Medicine and Pharmacy of Craiova, 200349 Craiova, Romania; adrianungureanu90@yahoo.com; 2 Department of Cardiology, Emergency County Hospital of Craiova, 200642 Craiova, Romania; eugen.tieranu@umfcv.ro (E.Ț.); cristiana.moise10@gmail.com (G.C.M.); 3 Department of Physiology, University of Medicine and Pharmacy of Craiova, 200349 Craiova, Romania; 4 Department of Cardiology, University of Medicine and Pharmacy of Craiova, 200349 Craiova, Romania; 5 Department of Pathophysiology, University of Medicine and Pharmacy of Craiova, 200349 Craiova, Romania; cristina.negroiu@yahoo.ro; 6 Department of Cardiology, University and Emergency Hospital, 050098 Bucharest, Romania; radu-mitrut@yahoo.co.uk; 7 Department of Cardiovascular Surgery, University of Medicine and Pharmacy of Craiova, 200349 Craiova, Romania; dr.raicea.victor@gmail.com; 8 Department of Cardiology, Faculty of Medicine, Carol Davila University of Medicine and Pharmacy, 050474 Bucharest, Romania; 9 Department of Internal Medicine, University of Medicine and Pharmacy of Craiova, 200349 Craiova, Romania; paulmitrut@yahoo.com

**Keywords:** left bundle branch area pacing, right ventricular septal pacing, new-onset atrial fibrillation, mildly reduced ejection fraction

## Abstract

**Background/Objectives:**Pacing treatment of bradyarrhythmias is both to reduce symptoms and to prevent syncope and sudden cardiac death. The aim of our study was to analyze left bundle branch area pacing (LBBAP) in the prevention of new-onset AF and the improvement of echocardiographic parameters in patients with mildly reduced left ventricular ejection fraction (LVEF) compared to patients with bradyarrhythmias but preserved LVEF who underwent mid-septal right ventricular pacing. **Methods**: This research was structured as a retrospective observational cohort study that included 186 patients with LBBAP and 186 patients with RVP, enrolled for 3 years until March 2024 with a follow-up time of 1 year. The primary endpoint of our study was new-onset atrial fibrillation after pacemaker implantation. The secondary endpoint was the improvement of echocardiographic parameters. **Results**: We observed in the LBBAP group a mean QRS complex duration of 108.7 ± 8.83 ms (after pacemaker implantation), compared to a much longer duration in the RVP group (143.8 ± 9.851 ms, *p* = <0.0001). At 1 year of follow-up, 22 (11.82%) patients in the RVP group were diagnosed with new-onset atrial fibrillation, compared to 6 (3.22%) patients out of 186 included in the LBBAP group (*p* = 0.0017). Regarding LVEF, at follow-up, RVP patients had a decrease in LVEF compared to those in the LBBAP group who had an improved LVEF (54.54 ± 3.77%, *p* < 0.0001). **Conclusions**: LBBAP both prevents the onset of atrial fibrillation and improves echocardiographic parameters, especially left ventricular ejection fraction, thus contributing to significantly reducing the risk of developing/worsening advanced heart failure through pacing-induced cardiomyopathy.

## 1. Introduction

The treatment goal for atrioventricular block is both to reduce symptoms and to prevent syncope and sudden cardiac death. First-degree atrioventricular block is generally asymptomatic [1]. On the other hand, high-grade and complete atrioventricular blocks frequently cause syncope and intense dizziness, especially those with paroxysmal onset [1]. In the case of chronic AV blocks, heart failure is more common than the appearance of other symptoms. Therefore, pacing is the only option that can improve the clinical condition of patients with atrioventricular blocks and an indication for pacing [1]. Right ventricular pacing has been a conventional therapy for bradycardia for more than 50 years [2]. However, in some patients, this method can lead to diminished cardiac function and, potentially, heart failure [3]. Furthermore, biventricular pacing has emerged over the past two decades as a viable treatment for patients with heart failure and certain conditions such as complete left bundle branch block, but it does not consistently produce the anticipated clinical outcome and has certain limitations [4,5,6,7].

The European Society of Cardiology (ESC) recently published a landmark consensus statement on conduction system pacing (CSP) [2], representing a notable advance in pacing therapy, confirming the practice of our electrophysiology laboratory that led to the results published in this article. Conduction system pacing, which involves the stimulation of the intrinsic electrical conduction pathways of the heart, has sparked increased interest in the search for alternative pacing strategies. His bundle branch pacing (HBP) was initially recognized for application in selected patients in the 2021 ESC Guidelines on Cardiac Pacing and Cardiac Resynchronization Therapy [1]. Recently, left bundle branch block pacing has been gaining popularity due to its perceived ease of implantation and improved electrical performance [8,9,10,11]. In patients with right ventricular pacing (RVP), electromechanical dyssynchrony is the main cause of adverse clinical outcomes [12,13]. Thus, there are several clinical trials or studies that have demonstrated that patients with RVP (especially with a high percentage) have a significantly increased risk of developing heart failure (HF) and new-onset atrial fibrillation (AF) [14,15,16]. Compared with RVP, LBBAP may improve the electrical performance, and therefore the mechanical performance of the heart, thereby reducing the occurrence of HF [17,18] and the occurrence of atrial fibrillation [12] in patients with left ventricular ejection fraction (LVEF) over 50%. A group of patients, not rarely encountered in clinical practice, those with LVEF between 40–49% classified as HF with mildly reduced ejection fraction (HFmrEF) [19] and bradyarrhythmias who underwent LBBAP, have not been analyzed regarding the occurrence of new-onset AF and improvement in EF.

The aim of our study was to analyze left bundle branch area pacing (LBBAP) in patients with bradyarrhythmias and mildly reduced ejection fraction, in terms of preventing new-onset atrial fibrillation (AF) and improving ejection fraction (EF), compared to patients with bradyarrhythmias but preserved EF who underwent right ventricular mid-septal pacing.

## 2. Materials and Methods

### 2.1. Study Design and Patient Selection

This research was structured as a retrospective observational cohort study and was conducted at the Emergency County Hospital of Craiova, affiliated with the University of Medicine and Pharmacy of Craiova, Romania. The research was conducted according to the principles enunciated in the Declaration of Helsinki and was approved by the Ethics Committee of the University of Medicine and Pharmacy of Craiova, number 108/01.03.2024. Patients provided written informed consent for the implantation procedure and the subsequent use of data for the purpose of scientific studies.

All patients with bradyarrhythmias eligible for the implantation of a dual-chamber pacemaker according to current guidelines [1,20,21] were consecutively enrolled to avoid bias, provided that they did not have a history of atrial fibrillation. Two groups of patients were conceived, those with LBBAP and with RVP (mid-septal), each group finally comprising 186 patients, with the enrollment period between March 2021 and March 2024 and a follow-up period of at least 1 year.

**Figure 1:**
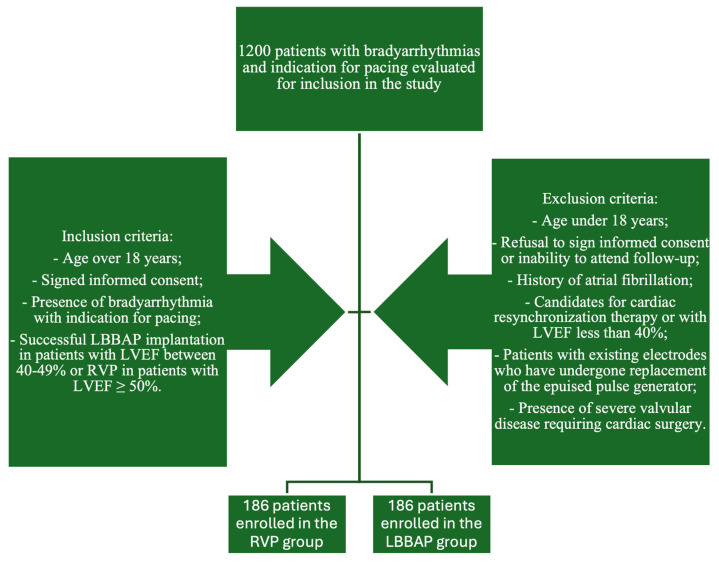
Study design.

The pacing strategies were established by the operators based on current practices. The LBBAP group included all patients who had undergone successful LBBAP procedures and who were at least 18 years old but with mildly reduced LVEF (40–49%), while the RVP group was formed by patients who received mid-septal pacing. The implantation method was decided based on LVEF. In our clinic, all patients are evaluated echocardiographically before the implant. The patients with LVEF over 50% were included in the RVP group, while patients with LVEF between 40 and 50% were included in the LBBAP group. We consecutively enrolled the patients, retrospectively. During patient enrollment, the manner of patient inclusion was 1:1; each time we enrolled an LBBAP implant, we subsequently included a patient with RVP. The exclusion criteria were established as follows: persons under 18 years of age; persons who refused to provide written informed consent or to participate in regular clinic check-ups; patients with a history of atrial fibrillation or previous trans-catheter ablations for atrial fibrillation; candidates for cardiac resynchronization therapy or with LVEF less than 40%; patients with existing leads who underwent replacement of the worn-out pulse generator; patients with severe valvular disease requiring cardiac surgery. The study design is shown in Figure 1.

### 2.2. Implant Procedure

**Figure 2:**
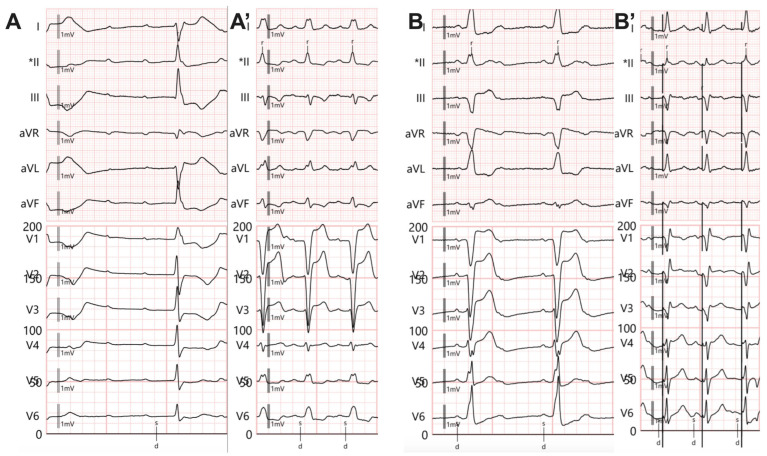
Twelve-lead electrocardiogram. (**A**) Complete atrioventricular block with infrahisian escape rhythm with right bundle branch block appearance. (**A’**) Right ventricular pacing, QRS morphology with left bundle branch block appearance, and prolonged QRS duration. (**B**) Complete atrioventricular block with infrahisian escape rhythm with complete left bundle branch block appearance. (**B’**) Left bundle branch area pacing (LBBAP) with R’ wave in V1 and LVATV6 = 71 ms. * The sensing channel.

For RV pacing, we used an active fixation lead that was placed in contact with the right ventricular septum in the middle region, using a preformed stylet. The fixation was performed in the anteroposterior view, and a 30° left anterior oblique view was applied to confirm the accuracy of the lead position, as previously described [20]. The paced QRS (pQRS) morphology was left bundle branch block with an intermediar axis as shown in Figure 2A,A’.

LBBAP was performed in all patients using the SelectSecure 3830 pacing lead and dedicated C315 His sheath (Medtronic, Minneapolis, MN, USA), as previously described [21]. The sheath and RV lead were positioned adjacent to the basal right ventricular septum, using anatomical and radiological criteria, with the initial positioning being performed in the RAO projection (15–30 degrees) using the tricuspid annulus as a radiological landmark to establish the depth in the sagittal plane and positioning in the mid-septal/mid-inferior region in the frontal plane. In selected cases, a septal ventriculography with contrast medium was required to establish anatomical landmarks and the contact of the support catheter with the ventricular septum. The lead was positioned by transseptal crossing, using 10–25 complete rotations depending on the situation (septal thickness, degree of fibrosis, initial contact, or crossing vector). During lead advancement, the QRS morphology was meticulously analyzed in dynamics during septal crossing by pacing in unipolar configuration (distal electrode–subclavicular pocket). Initially, the depth of the septal crossing was assessed by injecting contrast medium through the support catheter in the 30 degree LAO projection, an image that successfully marks the right border of the interventricular septum and prevents lead perforation into the left ventricle. The risk of septal perforation was reduced by monitoring the dynamics of the stimulation impedance (sudden drop in impedance by over 20% or to an absolute value < 450 ohm intraprocedural), the amplitude of the intracavitary signal, and the morphology of the local unipolar signal (QS aspect suggesting complete septal perforation). After 30–40 procedures performed in our laboratory, the need for all evaluation methods at each procedure (ventriculography, catheter stability, septal depth, pacing, and detection parameters) has decreased significantly. In most cases (59–96%) using radiological criteria for positioning, morphology, and duration of the pQRS complex, LVATV6 < 80 ms (left ventricular activation time in lead V6) demonstrated the presence of fixation beats during LBBAP, in cases [22,23,24].

**Figure 3:**
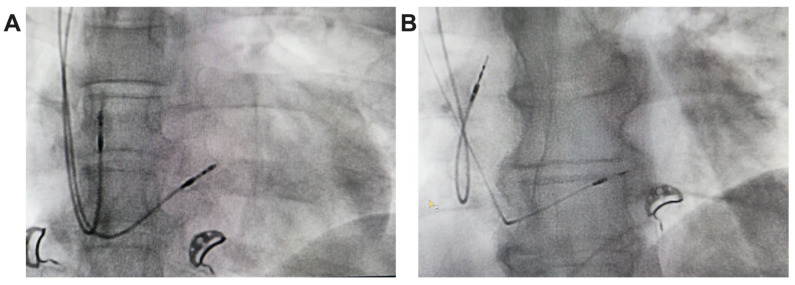
Radiological images, postero-anterior view, from the implant of a pacemaker with ventricular lead for RVP (**A**) and ventricular lead for LBBAP (**B**).

In unipolar pacing, left bundle branch area pacing (LBBAP) is characterized by a terminal r/R wave in lead V1 (Figure 2B,B’) or right bundle branch block (RBBB), accompanied by one of the following criteria for left bundle branch pacing: a short and consistent left ventricular activation time (LVAT from stimulus) below 80 ms at both high and low output in lead V6; the presence of the LBB potential; or a QRS transition during threshold testing or programmed ventricular pacing (from nonspecific LBBAP to specific LBBAP) [21]. Comparative fluoroscopic images for the two implant techniques/methods analyzed in our study are shown in Figure 3.

The Endurity (originally St Jude, now Abbot, CA, USA) or Vitatron G70A2 (Changi South, Singapore) dual-chamber pacemakers were equipped with advanced diagnostic features specifically designed for the automatic diagnosis and monitoring of AF.

### 2.3. Data Collection and Follow-Up

The baseline characteristics were collected, including demographic data (age, gender), comorbidities (hypertension, diabetes mellitus, coronary heart disease, NYHA heart failure class), ECG (QRS duration before the procedure), and echocardiographic parameters (left ventricular ejection fraction, LVEF; left ventricular end-diastolic diameter, LVEDD; left atrial volume, LAV; left atrial ejection fraction, LAEF) as well as biological parameters such as hemoglobin or creatinine. After discharge, all patients were monitored at 1, 3, 6, and 12 months, and annually thereafter. At the follow-up visits, AF-related data were uploaded and analyzed. This included keeping a diary of AF episodes, capturing electrograms (EGMs) and markers associated with stored episodes, recording the number and duration of episodes, and measuring heart rate before and at the onset of the arrhythmia. These regular follow-up visits were intended to monitor the progression and characteristics of AF in each patient. If no AF episodes occurred during follow-up, the patient was censored at the last follow-up or at death. We also monitored the percentage of atrial and ventricular pacing, impedance, R-wave amplitude, and the pacing threshold for the ventricular lead. Readmissions due to heart failure events were very rare among our patients (but 2 cases in the LBBAP group and 5 patients in the RVP group).

### 2.4. Study Endpoints

The primary endpoint of our study was the occurrence of atrial fibrillation after pacemaker implantation. The secondary endpoint was an improvement in left ventricular ejection fraction. New-onset AF was defined as episodes of atrial fibrillation detected by the device lasting at least 30 s on the intracardiac electrogram (usually the device automatically switching to DDI mode at an atrial heart rate above 180 beats per minute), or the diagnosis was made by performing a 12-lead surface ECG when the patient requested it or during routine check-ups.

### 2.5. Statistical Analysis

Continuous variables in this study were presented as mean ± standard deviation. Group means were compared using Student’s *t* test. Categorical variables were expressed as frequency or percentage and compared using the χ^2^ test or Fisher’s exact test. The Kaplan–Meier survival curve and log-rank test were used to estimate cumulative event rates for all patients enrolled in our study. A *p* value < 0.05 was considered statistically significant. All statistical analyses were performed with GraphPad Prism 10 (Graphpad Software, San Diego, CA, USA).

## 3. Results

### 3.1. Baseline Characteristics of All Enrolled Patients

The baseline characteristics of the study population are shown in Table 1. We did not observe statistically significant differences in terms of gender, age, smoking status, hypertension, diabetes mellitus, level of hemoglobin concentration, and level of creatinine. The first difference that we observed was in terms of NYHA functional class. Patients in the LBBAP group were from the beginning in a larger number, n = 35 (18.82%), with heart failure phenomena compatible with NYHA functional class III/IV vs. 19 (10.22%) patients in the RVP group who were classified at study enrollment in NYHA functional class I/II (*p* = 0.0266). Furthermore, in the LBBAP group there were 24 (12.90%) patients with coronary artery disease, compared to 10 (5.38%) patients in the RVP group who had coronary artery disease (*p* = 0.0182). Regarding echocardiographic parameters analyzed in our study, patients in the LBBAP group had a smaller LVEF (42.62 ± 2.34% versus 55.43 ± 3.25% for the RVP group, *p* = <0.0001), a larger left ventricular end-diastolic diameter (48.52 ± 4.52 mm versus 45.23 ± 4.03 mm for the RVP group, *p* = <0.0001), a larger left atrial volume (87.39 ± 30.98 mL versus 75.00 ± 21.07 mL for the RVP group, *p* = <0.0001), as well as a lower left atrial ejection fraction (45.30 ± 9.09% versus 49.31 ± 7.67% for the RVP group, *p* = <0.0001).

### 3.2. Radiation Dosage and Procedural Duration

**Figure 4:**
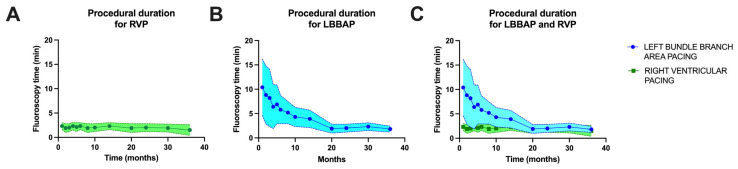
(**A**) Fluoroscopy time over the three years of enrollment in the right ventricular pacing (RVP) group of patients. (**B**) Fluoroscopy time over the three years of enrollment in the left bundle branch area pacing (LBBAP) group of patients. (**C**) Overlap of fluoroscopy time over the three years of enrollment for the two patient groups.

**Figure 5:**
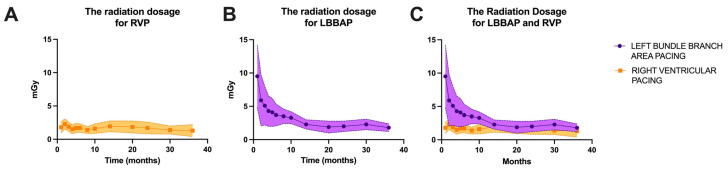
(**A**) Radiation dosage (milligray, mGy) over the three years of enrollment in the right ventricular pacing (RVP) group of patients. (**B**) Radiation dosage time over the three years of enrollment in the left bundle branch area pacing (LBBAP) group of patients. (**C**) Overlap of radiation dosage over the three years of enrollment for the two patient groups.

In our laboratory, probably similar to most of the pacing and invasive cardiac electrophysiology centers, the main goal is to minimize the duration of the procedure and radiation dosage. Given that at the beginning of the learning curve for left bundle branch area pacing in our laboratory, in 2021, the average duration of a procedure was 10.4 ± 5.8 min, while for right ventricular pacing, the average duration of a procedure was 2.3 ± 0.7 min (*p* < 0.0001). After three years of practice and more than 186 LBBAP procedures, the fluoroscopy times have become almost equal, as shown in Figure 4 (after three years of practice, LBBAP had an average fluoroscopy time of 1.8 ± 0.6 min versus 1.5 ± 1.1 min for RVP, *p* = 0.0733). Regarding radiation dosage, the observations were similar, with a radiation dosage of 9.5 ± 4.7 mG at the beginning of LBBAP practice compared to 1.8 ± 0.6 mGy for LBBAP versus 1.3 ± 0.9 mGy for RVP (*p* = 0.0601), as seen in Figure 5.

### 3.3. Electrocardiographic Parameters

**Figure 6:**
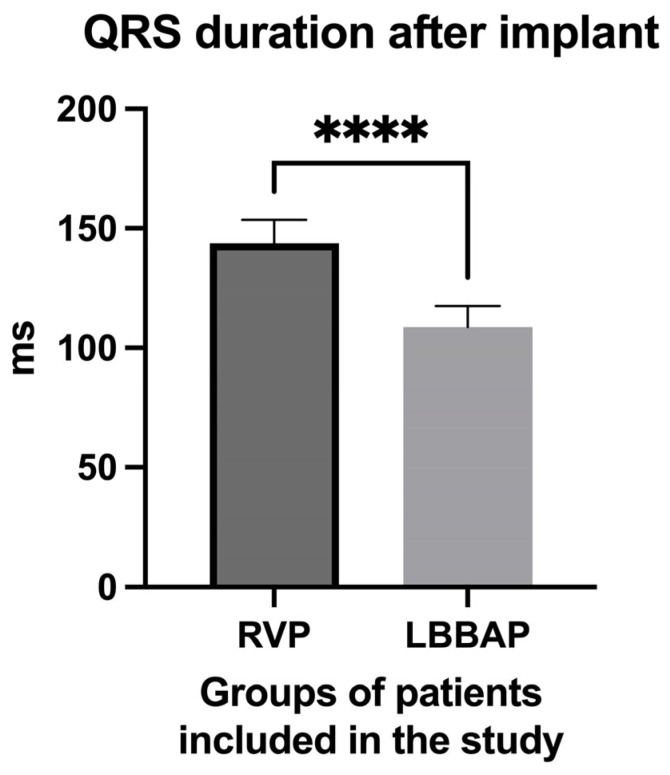
QRS complex duration in the group of patients with right ventricular pacing (RPP) after pacemaker implantation compared to the QRS complex duration in the group of patients with left bundle branch area pacing (LBBAP). **** extremely high statistical significance.

The most important electrocardiographic parameter used in our study was the QRS complex duration. We observed in the LBBAP group of patients a mean QRS complex duration of 108.7 ± 8.83 ms (Figure 6) after pacemaker implantation, compared with a much longer duration in the RVP group (143.8 ± 9.851 ms, *p* = <0.0001), although in this latter group of patients, only the patients who had the right ventricular lead implanted only mid-septal and not at the right ventricular apex or in other completely non-physiological areas were included.

### 3.4. New-Onset Atrial Fibrillation

**Figure 7:**
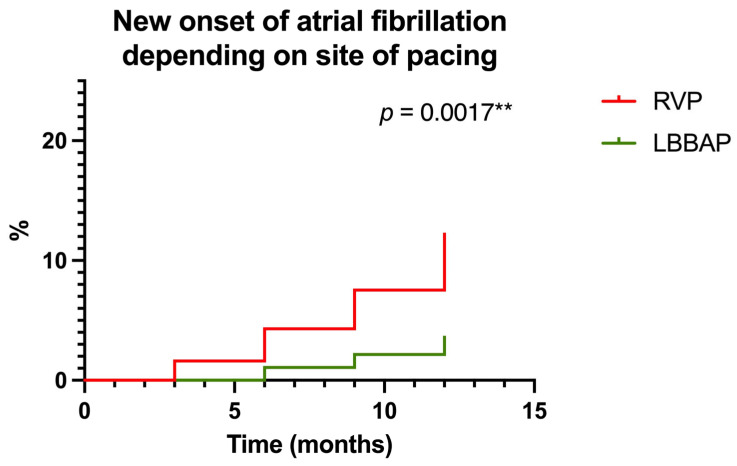
Probability of new-onset atrial fibrillation according to the pacing area (right ventricular pacing—RVP versus left bundle branch area pacing—LBBAP) at one-year follow-up. ** highly statistically significant.

One of the main endpoints of our study was to analyze new-onset atrial fibrillation in the two groups of patients (RVP versus LBBAP) during the 1-year follow-up period post-pacemaker implantation. Thus, we observed that at 1-year of follow-up, 22 (11.82%) patients in the RVP group were diagnosed with new-onset atrial fibrillation, compared to 6 (3.22%) patients out of the 186 included in the LBBAP group. Thus, for RVP, a Hazard Ratio of 3.321 (95% CI of ratio 1.571 to 7.017) was recorded compared to LBBAP where a Hazard Ratio of 0.3011 (95% CI of ratio 0.142 to 0.636, *p* = 0.0017) was recorded, as shown in Figure 7.

### 3.5. Echocardiographic Outcomes

**Figure 8:**
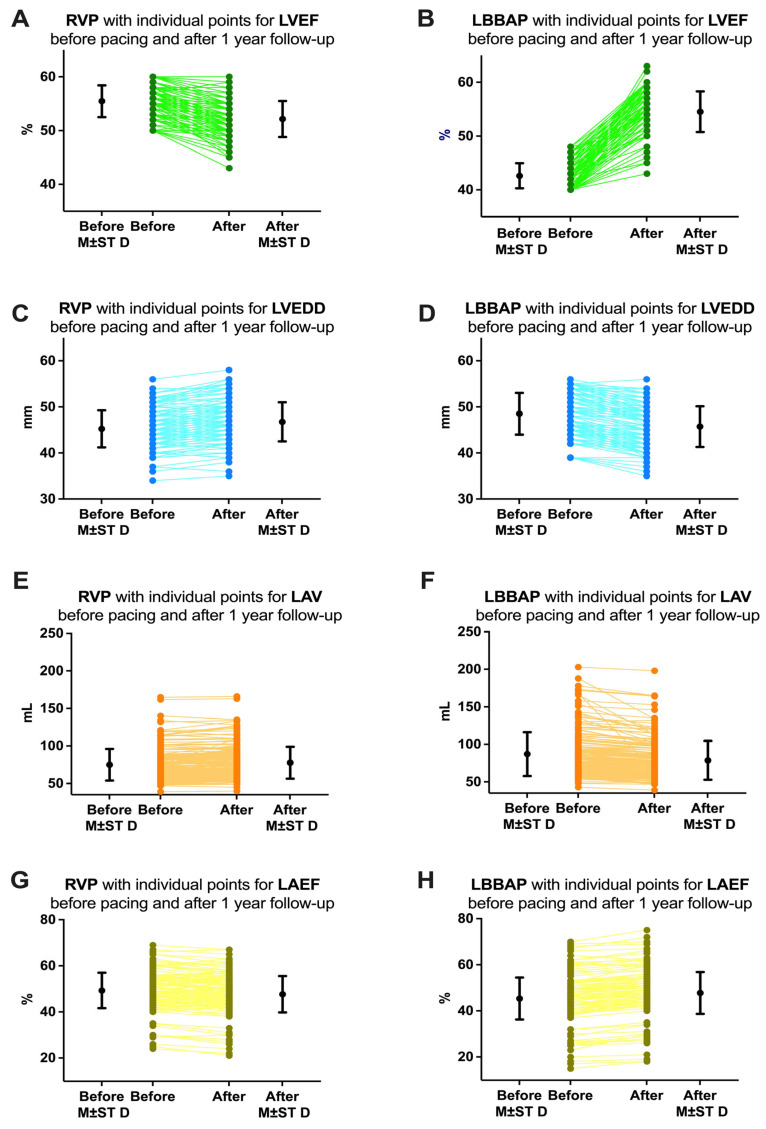
Variations of echocardiographic parameters analyzed in our study before pacemaker implantation and at 1 year of follow-up. (**A**) Left ventricular ejection fraction (LVEF) in patients with right ventricular pacing (RVP) and (**B**) in patients with left bundle branch area pacing (LBBAP). (**C**) Left ventricular end-diastolic diameter (LVEDD) in patients with RVP and (**D**) in patients with LBBAP. (**E**) Left atrial volume (LAV) in patients with RVP and (**F**) in patients with LBBAP. (**G**) Left atrial ejection fraction (LAEF) in patients with RVP and (**H**) in patients with LBBAP. M ± ST D = mean and standard deviation.

The first echocardiographic parameter analyzed in our study was the left ventricular ejection fraction (LVEF). Depending on this parameter, we also determined the pacing mode/area, whether we practiced right ventricular pacing or left bundle branch area pacing. In the RVP group, we included all patients who had LVEF above 50% before pacemaker implantation (55.43 ± 3.25%). Although at 1 year of follow-up these patients did not show a clinical difference in LVEF as it remained above 50% for most patients (52.16 ± 3.33), from a statistical point of view the difference was significant (*p* < 0.0001, Figure 8A). On the other hand, in the LBBAP group, if initially LVEF was slightly reduced (42.62 ± 2.34%), at one year of follow-up the patients showed improved LVEF (54.54 ± 3.77%, *p* < 0.0001, Figure 8B). Regarding the left ventricular end-diastolic diameter (LVEDD), reverse variations were observed. Before pacemaker implantation, patients in the RVP group had an LVEDD of 45.23 ± 4.03 mm, and patients in the LBBAP group had an LVEDD greater than 48.52 ± 4.52 mm. After one year of follow-up, patients in the RVP group had an increase in LVEDD (46.77 ± 4.24 mm, *p* = 0.0004, Figure 8C), while patients in the LBBAP group had a decrease in LVEDD to 45.73 ± 4.42 mm (*p* < 0.0001, Figure 8D). Regarding left atrial volume (LAV), before implantation, patients in the RVP group had an LAV of 75.00 ± 21.07 mL, and patients in the LBBAP group had an LAV greater than 86.98 ± 29.19 mL. After one year of follow-up, patients in the RVP group had a non-significant increase in LAV (77.72 ± 21.23 mL, *p* = 0.2156, Figure 8E), while patients in the LBBAP group had a decrease in LAV to 78.69 ± 25.83 mL (*p* = 0.0040, Figure 8F). Finally, we analyzed the left atrial ejection fraction (LAEF) and found that before implantation, patients in the RVP group had an LAEF of approximately 49.31 ± 7.67%, while for patients in the LBBAP group, the LAEF was much lower at 45.30 ± 9.09% (*p* < 0.0001). After the implant at 1 year of follow-up, the patients in the RVP group had a decrease in LAEF to 47.65 ± 7.88% (*p* = 0.0396, Figure 8G), while for the patients in the LBBAP group, the LAEF was increased to 47.76 ± 9.07% (*p*= 0.0093, Figure 8H).

### 3.6. Follow-Up of Pacing Parameters

We followed up on both the atrial and ventricular pacing percentages, the impedance of the pacing leads, the R wave amplitude, and the pacing threshold. The follow-up data on these parameters are included in Table 2. It is worth mentioning that regarding the percentage of atrial pacing, no statistically significant differences were recorded, with both patient groups having an average atrial pacing percentage of approximately 25–20%. Regarding the percentage of ventricular pacing, it should be mentioned that we programmed the algorithms to minimize the percentage of pacing in the RVP patient group, while in the LBBAP group, we preferred a higher percentage of pacing. Regarding the impedance of the ventricular lead, we observed a lower impedance both at implantation and at follow-up in the LBBAP group compared to the RVP group. On the other hand, the amplitude of the R wave was greater in the LBBAP group compared to the RVP group.

## 4. Discussion

Our study had as its primary endpoint the identification of new-onset atrial fibrillation after pacemaker implantation in patients who underwent traditional right ventricular mid-septal pacing (RVP) versus left bundle branch area pacing (LBBAP). Secondary endpoints were represented by the improvement of echocardiographic parameters (especially left ventricular ejection fraction—LVEF—but also left atrial parameters such as volume or ejection fraction) in patients with mildly reduced LVEF (between 40 and 49%) before pacemaker implantation. On the one hand, this group of patients has not been analyzed from this perspective, and on the other hand, we are very pleased that the results of our study are in accordance with the recent expert consensus presented at the European Heart Rhythm Association (EHRA) congress in March 2025 [2].

Our group recently published another study in which it showed that heart failure or large left atrial volumes and areas, as well as much less studied factors such as the percentage of atrial/ventricular pacing or the presence of hepatic impairment, are predictors of the occurrence of atrial fibrillation (AF) in patients with cardiac pacemakers, but the patients included in that study had an LVEF above 50% [16]. Furthermore, recent studies of new-onset AF in patients with pacemaker implants also included patients who had an LVEF above 50% [12]. We included and analyzed a group of patients with a mildly low LVEF (between 40 and 49%).

The negative impact of right ventricular pacing on cardiac function and the risk of new-onset atrial fibrillation have been extensively documented in several studies [12,15,16,17,25,26,27]. In the MOST trial, the incidence rate of atrial fibrillation in patients implanted with pacemakers was 21% among patients with sick sinus syndrome who were implanted with dual-chamber (DDD) pacemakers [12,14,15]. Subsequent studies have shown that the rate of ventricular pacing was associated with the incidence of persistent atrial fibrillation [12]. Therefore, reducing the rate of ventricular pacing represents a strategy to reduce the risk of atrial fibrillation. Patients with sick sinus syndrome may be at an increased risk of developing atrial fibrillation due to a degenerated sinoatrial node and the progressive degeneration of atrial tissue. However, for patients with sick sinus syndrome and no prior history of atrial fibrillation, the natural progression of the disease may be altered if they benefit from prompt pacemaker implantation to restore normal heart rhythms, atrioventricular conduction, and ventricular synchronization [12]. Therefore, given the impact of pacing therapy, the likelihood of developing atrial fibrillation in patients with sick sinus syndrome and no prior history of atrial fibrillation may not differ significantly from that in individuals with atrioventricular block [12]. His bundle pacing is theoretically the most physiological pacing modality, and it is associated with a reduced risk of mortality, hospitalization for heart failure, or upgrade to biventricular pacing compared with right ventricular pacing [28]. The favorable impact of His bundle pacing on reducing the risk of new-onset atrial fibrillation has been documented compared with right ventricular apical pacing [28,29]. Left bundle branch area pacing, an alternative physiologic pacing method to His bundle pacing, has advanced rapidly in recent years, and has been routinely practiced in our center since 2021. Compared with His bundle pacing, LBBAP has demonstrated a lower pacing threshold and better detection amplitude, comparable paced QRS duration, and a reduced risk of increased capture threshold or loss of capture [2]. Compared with RVP, LBBAP has demonstrated superior left ventricular electromechanical synchronization and a reduced incidence of hospitalization for heart failure or transition to biventricular pacing [17]. In another large prospective observational study, LBBAP emerged as an independent protective factor for the risk of mortality, the risk of hospitalization for heart failure, and upgrade to biventricular pacing compared with right ventricular pacing [30]. When the percentage of ventricular pacing is greater than 20%, LBBAP has a beneficial effect on hemodynamics compared with patients with right ventricular pacing [12]. Left atrial function is closely related to left ventricular (LV) mechanical function [31]. Right ventricular pacing significantly increases left ventricular electromechanical delay and left intraventricular dyssynchrony, resulting in increased left atrial volume before atrial contraction, a decreased volume, and reduced passive and total emptying fractions compared with His bundle stimulation [12]. Our study also showed that LBBAP reduces left atrial (LA) volume, improves LA ejection fraction, in parallel, obviously, with the improvement of left ventricular ejection fraction.

Detection of new-onset atrial fibrillation is particularly important in patients with pacemakers, especially in the context in which these patients usually have significant comorbidities that further complicate their therapeutic management [16,32,33,34]. Multiple clinical studies have shown that left atrial dilation initiates the electrical processes that cause atrial fibrillation. The correlation in patients with pacemakers and its effect on the occurrence of atrial arrhythmias has also been examined, revealing a significantly larger left atrial diameter in individuals with atrial fibrillation, a finding already established in previous research [34,35]. Surprisingly, patients in our study in the LBBAP group, although initially presenting with a smaller left atrial ejection fraction and larger volumes, nevertheless, after implantation, presented AF in a much lower proportion compared to patients who had these much smaller left atrial dimensions but were traditionally paced with right ventricular pacing. Numerous studies have demonstrated the presence of coronary artery disease (CAD) in cohorts of patients with pacemakers and atrial fibrillation [36]. However, our study failed to demonstrate the association between the risk of atrial fibrillation and coronary artery disease in these patients.

Even though the successes are evident in the case of LBBAP, it is still a relatively new procedure that requires validation, especially in terms of complications. A recent meta-analysis of 15 observational studies, including 2491 patients, demonstrated that LBBAP had significantly higher success rates versus His bundle pacing (91.1 vs. 80.9%; *p* < 0.001) [37]. Also, regarding complications, there were significantly fewer lead-related complications during follow-up, which included lead failure, inactivation due to high thresholds, and displacement (1.1 vs. 4.3%; *p* = 0.003). In addition, the meta-analysis also found no significant difference in lead displacement rates between LBBAP and traditional right ventricular pacing [37]. Although infections can occur in patients with LBBAP, just as in patients with traditional right ventricular pacing [38], recent studies show that the extraction procedure is safe and effective, with a low need for extraction tools and minimal complication [39,40,41,42,43,44].

Regarding the cost of LBBAP, it is almost double compared to RVP. However, given its benefits in patient outcomes, LBBAP is worth practicing, as the cost will be compensated by the reduction in hospitalizations for heart failure events or the treatment of comorbidities such as atrial fibrillation.

### Limitations of Our Study

Firstly, this study was retrospective and did not include a very large number of patients. Secondly, the study was conducted in a single center and is a non-randomized observational study. Furthermore, patients were included in the LBBAP group according to slightly reduced left ventricular ejection fraction (LVEF between 40 and 49%) but the duration of bradyarrhythmia could not be analyzed, which could have further decreased LVEF or could have caused an increase in left ventricular end-diastolic diameter and left atrial volume or could have caused a decrease in left atrial ejection fraction.

## 5. Conclusions

Our study clearly showed that left bundle branch area pacing should be the pacing method for patients with mildly reduced left ventricular ejection fraction. This method both prevents the occurrence of atrial fibrillation and improves echocardiographic parameters, especially the left ventricular ejection fraction, thus contributing to a significant reduction in the risk of developing/worsening advanced heart failure due to pacing-induced cardiomyopathy. Moreover, in the near future, this method will probably become the standard of pacing considering its feasibility and the major benefits it brings.

## Figures and Tables

**Table 1:** Clinicopathological characteristics of patients at study enrollment.

Variables		RVP (n = 186)	LBBAP (n = 186)	*p* Value
Gender	Male	100 (53.76%)	113 (60.75%)	0.2085 ^#^
	Female	86 (46.24%)	73 (39.25%)	
Indication for implant	Sick sinus syndrome	39 (20.97%)	54(29.03%)	0.0934 ^#^
	AV block	147 (79.03%)	132 (70.97%)	
Age (years)		71.03 ± 9.08	70.80 ± 8.96	0.8006 *
Smoke	Yes	29 (15.59%)	37 (19.89%)	0.3421 ^#^
Hypertension	Yes	102 (48.34%)	109 (51.66%)	0.5302 ^#^
Heart failure	NYHA I/II	167 (89.78%)	151 (81.18%)	0.0266 ^#^
	NYHA III/IV	19 (10.22%)	35 (18.82%)	
Coronary artery disease	Yes	10 (5.38%)	24 (12.90%)	0.0182 ^#^
Diabetes mellitus	Yes	63 (33.87%)	60 (32.26%)	0.8256 ^#^
Hemoglobin (g/dL)		13.72 ± 1.637	13.63 ± 1.741	0.5958 *
Creatinine (mg/dL)		1.209 ± 0.4234	1.275 ± 0.4848	0.1586 *
Echo parameters				
LVEF (%)		55.43 ± 3.25	42.62 ± 2.34	<0.0001 *
LVEDD (mm)		45.23 ± 4.03	48.52 ± 4.52	<0.0001 *
LAV (mL)		75.00 ± 21.07	87.39 ± 30.98	<0.0001 *
LAEF (%)		49.31 ± 7.67	45.30 ± 9.09	<0.0001 *

* *t* test. ^#^ Fisher’s exact test.

**Table 2:** Follow-up of pacing parameters for 1 year post-implant.

The Time of Assessment	Parameters	RVP Group (n = 186)	LBBAP Group (n = 186)	*p* Value *
At implant	Impedance (ohm)	579.8 ± 73.93	547.5 ± 66.89	<0.0001
	Pacing threshold (V/0.4 ms)	0.7245 ± 0.1792	0.6008 ± 0.1409	<0.0001
	R-wave amplitude (mV)	10.82 ± 4.427	12.98 ± 4.438	<0.0001
1-month follow-up	Atrial pacing (%)	28.44 ± 32.99	25.93 ± 31.84	0.4566
	Ventricular pacing (%)	70.73 ± 28.80	80.54 ± 24.10	0.0004
	Impedance (ohm)	580.1 ± 75.00	523.5 ± 72.31	<0.0001
	Pacing threshold (V/0.4 ms)	0.7419 ± 0.1926	0.6129 ± 0.1645	<0.0001
	R-wave amplitude (mV)	9.923 ± 4.361	13.31 ± 4.355	<0.0001
3-month follow-up	Atrial pacing (%)	29.19 ± 32.80	26.77 ± 31.62	0.4684
	Ventricular pacing (%)	71.52 ± 30.20	83.61 ± 22.73	<0.0001
	Impedance (ohm)	565.2 ± 76.58	504.1 ± 72.65	<0.0001
	Pacing threshold (V/0.4 ms)	0.7567 ± 0.2037	0.6397 ± 0.1799	<0.0001
	R-wave amplitude (mV)	9.805 ± 4.381	13.12 ± 4.415	<0.0001
6-month follow-up	Atrial pacing (%)	32.24 ± 33.41	29.85 ± 32.02	0.4822
	Ventricular pacing (%)	70.70 ± 30.59	82.79 ± 23.26	<0.0001
	Impedance (ohm)	566.2 ± 75.99	478.0 ± 82.12	<0.0001
	Pacing threshold (V/0.4 ms)	0.7648 ± 0.2193	0.6359 ± 0.2015	<0.0001
	R-wave amplitude (mV)	9.199 ± 4.344	13.15 ± 4.198	<0.0001
12-month follow-up	Atrial pacing (%)	30.45 ± 32.85	28.01 ± 31.57	0.4654
	Ventricular pacing (%)	69.76 ± 30.54	81.73 ± 23.33	<0.0001
	Impedance (ohm)	558.5 ± 77.43	442.8 ± 82.96	<0.0001
	Pacing threshold (V/0.4 ms)	0.7917 ± 0.2395	0.6720 ± 0.2368	<0.0001
	R-wave amplitude (mV)	9.046 ± 4.112	13.82 ± 4.252	<0.0001

* The *p* value in the *t*-test.

## Data Availability

Data are contained within the article or available upon request from the corresponding authors.
